# Limited Knowledge of Chronic Kidney Disease among Type 2 Diabetes Mellitus Patients in India

**DOI:** 10.3390/ijerph16081443

**Published:** 2019-04-23

**Authors:** Salman Hussain, Anwar Habib, Abul Kalam Najmi

**Affiliations:** 1 Department of Pharmaceutical Medicine (Division of Pharmacology), School of Pharmaceutical Education and Research, Jamia Hamdard, New Delhi 110062, India; salmanpharma@gmail.com; 2 Department of Medicine, Hamdard Institute of Medical Sciences and Research, Jamia Hamdard, New Delhi 110062, India; 3 Department of Pharmacology, School of Pharmaceutical Education and Research, Jamia Hamdard, New Delhi 110062, India; aknajmi@jamiahamdard.ac.in

**Keywords:** chronic kidney disease, diabetes, hypertension, diabetic kidney disease, epidemiology, India, public health

## Abstract

Diabetes and hypertension are the two major causes of chronic kidney disease (CKD). Epidemiological studies have found poor knowledge about the CKD among the general population. Hence, this study aimed to assess the awareness of CKD among type 2 diabetes mellitus (T2DM) patients in India. Patients with confirmed T2DM were included in the study. Patients receiving dialysis or with a history of a kidney transplant were excluded. A validated questionnaire was used to assess knowledge about CKD. Demographic characteristics were presented using descriptive statistics and trends in groups were calculated using the chi-square test. Statistical analysis was performed using SAS v9.4. A total of 323 patients completed the study. The mean age of the patients was 56 ± 11.25 years, and 51.7% were female. Only 33.43% of the patients correctly identified diabetes and hypertension as risk factors for CKD, while 44.27% were aware of the kidney’s function. Statistically significant associations were observed between kidney disease knowledge and education status (*p* = 0.004), socioeconomic status (*p* = 0.000), and income status (*p* = 0.003). No association was observed between the knowledge about CKD and age, gender, hypertension stages, CKD stages, duration of diabetes as well as hypertension and co-morbidities. Based on the results of this study, we found poor knowledge of CKD among Indian T2DM patients. The government should start a CKD awareness programme to deal with this rising co-morbid condition.

## 1. Introduction

Diabetic kidney disease (DKD), also known as diabetic nephropathy, is a common microvascular complication of type 2 diabetes mellitus (T2DM). DKD is considered a major public health problem for both the patient and the healthcare system. Diabetes and hypertension are two major risk factors of chronic kidney disease (CKD) [1]. Even pre-hypertension is associated with an increased risk of CKD, with a relative risk of 1.28 (95% CI: 1.13 to 1.44) [2]. Albuminuria and proteinuria are hallmarks for CKD, which is characterized by a decline in renal function [3,4]. Diabetes and hypertension are also the most common causes of end-stage renal failure, accounting for nearly 50% of cases [5,6,7]. In the United States (US), diabetes and hypertension are the leading causes of kidney failure in CKD patients [8]. CKD has high global prevalence, with rates reported between 11% to 13% [9]. The Start India Project, which assessed the prevalence of CKD among diabetic patients, has estimated that more than 40% of T2DM patients have CKD. Likewise, one in five hypertensive subjects has CKD [10]. According to the 2010 Global Burden of Disease study, diabetes and CKD were ranked 9th and 18th among the listed causes of overall global mortality [11]. DKD was also associated with higher mortality, morbidity, and healthcare expenditure [12,13], imparting high economic burden on DKD patients [14].

Real world studies have also suggested a significant and incremental increase in the economic burden for DKD patients [14,15]. A retrospective cohort study found that T2DM patients with co-morbid stage-4 CKD incur yearly costs of USD 33,162 for their treatment [16]. In another retrospective observational study, DKD was found to be significantly associated with higher costs of treatment, healthcare resource use, and risk of disease progression [17]. According to a report from the National Health Service (NHS), treating kidney disease costs more than skin, lung, and breast cancer combined [18]. The economic burden due to CKD can be reduced if patients are diagnosed at an early stage. In low- and middle-income countries (LMICs), the majority of the patients generally only become aware of their CKD status when they reach the end-stage of kidney failure and require dialysis [19]. Furthermore, due to the absence of appropriate healthcare coverage and limited access to renal replacement therapy, the treatment of end-stage renal disease (ESRD) becomes unaffordable in LMICs, and patients are bound to pay out-of-pocket [20]. Timely diagnosis and treatment are the most cost-effective and clinically appropriate public health strategies to deal with this condition.

Early identification and treatments for DKD have been shown to slow, stop, or even reverse the progress of the disease and the decline of kidney function [21,22]. Yet the majority of CKD cases are not identified early. Limited knowledge of CKD is the main barrier to the early diagnosis and prevention of disease progression [23]. Several epidemiological studies have reported about the limited knowledge of CKD among the general public [24,25]. A global cross-sectional study done in six regions (Eastern Asia, Southern Asia, Middle East, Africa, Eastern Europe, and Latin America) found only 6% of the public to be aware of CKD [26]. Similarly, the data provided by the Kidney Early Evaluation Program (KEEP) and the National Health and Nutrition Examination Survey (NHANES) revealed that only 9% of the DKD patients were aware of their CKD status [27]. A study by Chow et al. found there to be poor knowledge of CKD in the general public and suggested future studies in population of high-risk individuals [24]. In a resource-limited country like India, where there are only 6.49 physicians for every 10,000 people [28] and the cost of dialysis is unaffordable [19], awareness of the disease is of paramount importance and is considered to be the first step toward prevention. Therefore, this study aimed to evaluate the knowledge of CKD among T2DM patients in India.

## 2. Methodology

### 2.1. Study Setting

This cross-sectional study was conducted at the out-patient department of endocrinology, at Hakeem Abdul Hameed Centenary (HAHC) Hospital, Jamia Hamdard, New Delhi, India. The study period was from April 2017–May 2018. Before the initiation of the study, the study protocol was approved by the Jamia Hamdard Institutional Ethics Committee (JHIEC-2017-04/17), India. The study was conducted in full compliance with the Declaration of Helsinki guidelines [29] and written in accordance with the Strengthening the Reporting of Observational Studies in Epidemiology (STROBE) guidelines for reporting a cross-sectional study [30]. Written informed consent was provided voluntarily by the participants. Participants were assured of their confidentiality and the anonymity of their identity.

### 2.2. Study Population

Patient selection was carried out on the basis of pre-defined inclusion and exclusion criteria. Only participants that fulfilled all the following criteria were included in the study: (a) willingness to participate in the study by providing signed informed consent form; (b) patients aged 18 years or above of either sex; (c) patients who had confirmed T2DM as per their medical records. Participants were excluded from the study if they were receiving dialysis or had a history of a kidney transplant. Patients with incomplete interview information were also excluded.

Patients’ demographic characteristics like age, sex, marital status, substance use (smoking, drinking), and family history were recorded in the case record form. We used modified Kuppuswamy’s socioeconomic scale to assess the economic status of the patients. According to this scale, socioeconomic status was divided into five subscales (upper, upper middle, lower middle, upper lower, and lower) on the basis of occupation, education, and family income. The majority of the patients fell into two categories, lower or lower middle class, so the patients were categorized into two classes (i.e., lower class or middle class).

### 2.3. Questionnaire Used

The questionnaire used to assess the CKD awareness was adopted from Chow et al. [24]. The questionnaire consisted of seven questions covering aspects of anatomy (number of kidneys needed to lead a normal life), physiology (the function of a kidney), etiology (risk factors of CKD), presentation (symptoms of CKD, progression), resources availability, and treatment (please refer to the supplementary file for the questionnaire). Every correct answer was allocated one point, so that patients could score a maximum of seven points or a minimum of zero point. Patients were considered to have good knowledge if they scored ≥4 points and poor knowledge if they scored <4 points.

### 2.4. Clinical and Laboratory Analysis

Anthropometric parameters and blood pressure were recorded by the trained nurse personnel. Blood pressure was measured using Richter auscultatory sphygmomanometers at two times: one at 5 min of rest and another at sitting position. Mean systolic blood pressure (SBP) and diastolic blood pressure (DBP) were recorded. Patients’ blood pressure records were also reviewed for an accurate reflection of hypertension stage. Patients were classified as hypertensive if the mean SBP was ≥140 mmHg and DBP was ≥90 mmHg or if they had been previously prescribed antihypertensive medication. Patients were further categorized as prehypertensive (SBP 120–139 mmHg or DBP 80–89 mmHg), stage-I hypertensive (SBP 140–159 mmHg or DBP 90–99 mmHg), and stage II hypertensive (SBP ≥ 160 mmHg or DBP ≥ 100 mmHg) [31]. Similarly, T2DM was defined on the basis of their medical record or fasting plasma glucose and/or glycated hemoglobin (HbA1c) level or as per the American Diabetes Association (ADA) guidelines (random blood sugar ≥ 200 mg/dL) [32]. To assure the accurate reflection of glycemic control, we also reviewed the patients’ HbA1c records. HbA1c level below 7 was considered a good glycemic control, and HbA1c ≥ 7 was considered a poor glycemic control. CKD was defined on the basis of kidney function, as determined by means of estimated glomerular filtration rate (eGFR). The Chronic Kidney Disease Epidemiology Collaboration (CKD-EPI) equation was used to calculate the eGFR (mL/min/1.73 m^2^) [33]. The National Kidney Foundation (NKF) classified CKD into six stages based on eGFR, where stage I refers to eGFR ≥ 90, stage II refers to eGFR 60–89, stage IIIa refers to eGFR 45–59, stage IIIb refers to eGFR 30–44, stage IV refers to eGFR 15–29, and stage V refers to eGFR < 15 [34]. Due to the small number of patients in CKD stage IIIa, IIIb, and IV, we included these classifications together under CKD stage III. The status of co-morbidities and duration of diabetes and hypertension were confirmed by physician, patients’ previous medical records, and current prescription. Literacy was self-reported by the patient.

Blood samples (5 mL) were collected for the routine test. The HbA1c test was performed by using a fully automated HPLC using a BIORAD testing system. Serum creatinine was determined by a modified Jaffe colorimetric method using a fully automated Siemens adiva-1800 chemistry analyzer (Siemens Healthcare Pvt Ltd., Mumbai, India). Blood glucose levels were determined by using a fully automated Roche Cobas 6000 analyzer (Roche, Mannheim, Germany). The entire tests were performed in the central pathology lab of Hamdard Institute of Medical Sciences and Research.

### 2.5. Statistical Analysis

Demographic characteristics were presented using descriptive statistics. Categorical variables were presented by frequency and percentages. Differences and association were computed using the chi-square test and multiple logistic regression. We considered good knowledge (≥4 scores) and poor knowledge (<4 scores) as the dependent dichotomous variable and associations were computed using the chi-square test. Similarly, we considered correct and incorrect responses of every question (domains) as the dependent dichotomous variable and associations with the demographic characteristics and clinical parameters were calculated using the chi-square test. A *p* value < 0.05 was considered statistically significant. All the statistical analyses were performed using SAS v9.4 (SAS Institute Inc: Cary, NC, USA).

## 3. Results

A total of 365 patients participated in the study, of which 42 were excluded, as they did not fulfill the inclusion criteria. The mean ± SD age of patients was 56 ± 11.25 years, and 51.7% were female. The majority (96.3%) was married and not covered by health schemes or health insurance and bound to pay out-of-pocket for the treatment. More than three-fourth of patients (77.7%) was of lower socioeconomic status and 63.5% were educated. Mean ± SD duration of diabetes was 10 ± 4.39 years and approximately 43.3% of the patients had a family history of diabetes. CKD stage III was prevalent in 34.4% (eGFR < 60 mL/min/1.73 m^2^) of patients, while 23.5% and 17.3% of patients had hypertension stage I and stage II, respectively. Demographic characteristics are presented in Table 1.

### 3.1. Patient’s Knowledge of CKD

**Figure 1:**
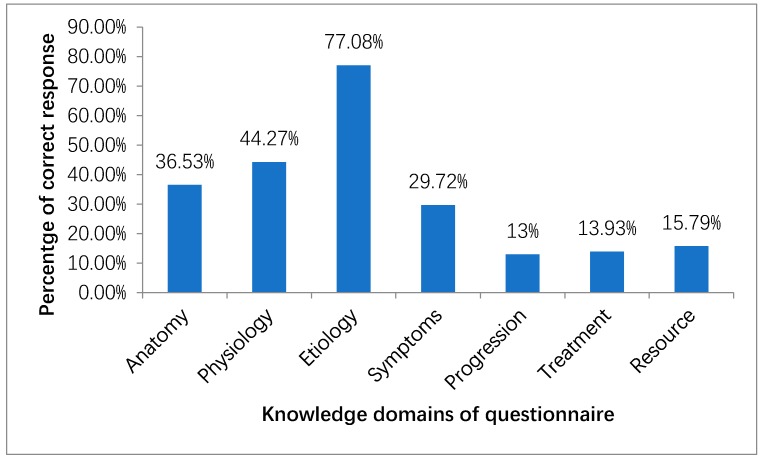
Percentage of patients with correct responses to the administered questionnaire.

Amongst seven domains of CKD knowledge, 118 patients (36.53%) correctly answered the anatomy part of the questionnaire that only one kidney is required to live a normal life. Less than half of the study population (44.27%) was aware that the filtration of waste products in the blood is the function of the kidney. More than three-fourth of the respondents (77.08%) correctly identified the risk factors of CKD in which only one-third (33.43%) of the respondents were aware of diabetes and hypertension as risk factors. Only 6.5% correctly identified hypertension, diabetes, and inherited condition as risk factors for the development of CKD. While 29.72% of the patients correctly identified symptoms of early kidney disease that might progress to kidney failure, only 4.95% of patients were aware that kidney disease could present without any symptoms. Likewise, a similar percentage of patients correctly identified that medications cannot cure CKD. Only 13.93% of patients were aware that dialysis treatment can be carried out both in a dialysis center and at home. Figure 1 shows the percentages of patients who correctly answered the questions.

Only 21.36% of respondents were found to have good knowledge (≥4 scores) of CKD, and the remaining had poor knowledge (<4 scores) of CKD (Table 2). Respondents who were literate, had a monthly family income more than 20,000 INR (Indian National Rupees), and belong to the middle class were found to have significantly good knowledge of CKD (all *p* < 0.05). No significant difference in knowledge score was observed among respondents stratified according to CKD stages, hypertension stages, BMI, gender, co-morbidities, family history, and duration of diabetes and hypertension (all *p* > 0.05).

### 3.2. Factors Influencing CKD knowledge

Being older (≥50 years), female gender, substance use (smoking, drinking), and occupation were non-significantly associated with CKD knowledge in all of the seven domains of the questionnaire. Patients who had a family history of diabetes were more likely to have a higher knowledge of CKD in all the domains except the treatment domain.

Patients belonging to the middle class were found to have a significantly higher knowledge of CKD in almost every domain of the CKD questionnaire (*p* < 0.05). Similarly, patients who had a monthly family income more than 20,000 INR were found to have a significantly higher knowledge of CKD (*p* < 0.05). Poor glycemic control patients were found to have a higher knowledge of the anatomy-related part of the questionnaire, with an odds ratio of 1.71 (95% CI: 1.04 to 2.80) as compared to good glycemic control patients (Table 3 and Table 4). CKD stage II and III patients had significantly higher knowledge in the physiology and disease progression parts of the questionnaire, respectively (*p* < 0.05), in comparison to the stage I CKD patients. A detailed description of the results is presented in Table 3 and Table 4. Multiple logistic regression analysis reveals that patients who were literate (adjusted odds ratio (AOR) 1.78 (95% CI: 1.30–2.36), *p* = 0.02) and had a higher family income (AOR 2.26 (95% CI: 1.66–3.14), *p* = 0.04) was found to have significantly good knowledge of CKD.

## 4. Discussion

It is evident from the literature that burden of diabetes is continuously increasing, which will contribute to the rising prevalence of CKD globally [9,10]. Published literature revealed that CKD can be reversible and preventable from its progression to end-stage kidney disease if it is diagnosed at an early stage [21,22]. Previous studies found poor knowledge of CKD among the general public, however, limited studies have assessed the knowledge of CKD among high-risk patients [23,24,25]. This was the first cross-sectional study assessing the knowledge of CKD among a high-risk population with diabetes and hypertension as co-morbid conditions. The current study revealed poor knowledge of CKD among these patients. Less than a quarter of the patients had good knowledge of CKD (knowledge score ≥ 4). Poor knowledge of CKD was also found in other studies conducted around the globe [24,35].

In this study, only one-third (33%) of the patients identified hypertension and diabetes as risk factors of CKD. A cross-sectional survey conducted among the non-CKD Malaysian population found half of the population (51.2%) to be aware of diabetes and hypertension as risk factors of CKD [24]. Variation in risk factor awareness could be due to the higher education level among the Malaysian population, as all (100%) the respondents were literate and 78.3% of the respondents had secondary or above secondary education levels [24]. Similarly, a cross-sectional telephonic survey among community-dwelling adults also revealed poor awareness of CKD and less than half of the population were aware of diabetes or hypertension as risk factors of CKD [35]. A lack of awareness about common risk factors like hypertension and diabetes is raising the alarm for the need for further action. Furthermore, this population is less likely to get proper screening, adhere to prescribed medication regimens, or take part in decision making, which may ultimately contribute to the rising prevalence of CKD and further progression of CKD to its end stages. Our study also found that patients who had a family history of diabetes or poor glycemic control were more likely to have a higher knowledge of CKD, including the risk factors. Higher knowledge among these patients could be possibly due to poor health status (uncontrolled diabetes), or patients who have heard of CKD are more likely to have better knowledge [36]. A study by Fezeu et al. [37] also concluded that patients whose relatives had a chronic condition are generally more aware (*p* < 0.001) of the disease. Patients belonging to the middle class or having higher family income were also found to have a higher knowledge of CKD. Family income is considered to be an important factor affecting the quality of life among non-dialysis CKD patients [38]. Previous studies also suggested that low income is associated with a higher prevalence of chronic conditions [39].

No significant difference in knowledge score was observed among respondents stratified according to CKD stages, hypertension stages, BMI, age, gender, and co-morbidities. The findings of Finkelstein et al. [40] also found no impact of age, gender, and disease on patients’ knowledge of CKD. However, they found that knowledge of CKD improved significantly as the CKD stage progressed, particularly in stage 5 CKD. Our result was contrary to that, as we did not find any significant improvement in knowledge on the basis of CKD stages. A possible reason for this could be due to the inclusion of non-dialysis (stage I to III CKD) patients only.

The study should be interpreted in light of certain limitations. Firstly, the findings of the study cannot be generalized to the entire CKD population, as the study was comprised only of patients from a single center. Secondly, the study was limited in regard to the selection of participants (selection bias); to overcome this, we consecutively selected the participants. There were several notable strengths of this study. Firstly, the study included T2DM and hypertensive patients and performed stratification based on CKD and hypertension stages. Secondly, the knowledge level was correlated with diabetes and hypertension status. The present study also highlighted the needs of CKD education among T2DM and hypertensive patients, since a lack of knowledge about these risk factors was reflected in the study that may have resulted in late referral to the nephrologist and poor participation in the decision-making process. Future studies are warranted to assess the CKD knowledge in a large population-based sample and to frame a CKD awareness model for high-risk patients as well as the general population in order to promote earlier diagnosis, better treatment, and innovative care.

## 5. Conclusions

We found poor knowledge of kidney disease among T2DM and hypertensive Indian patients. The government should start a CKD awareness programme to deal with this devastating co-morbid condition, which would help in achieving cost-effective prevention.

## Figures and Tables

**Table 1:** Background characteristics of the patients.

Variables	Male	Female	Total	*p* Value
Age groups				>0.999
≥50	93 (59.6%)	99 (59.3%)	192 (59.4%)	
<50	63 (40.4%)	68 (40.7%)	131 (40.6%)	
Marital Status				>0.999
Married	150 (96.2%)	161 (96.4%)	311 (96.3%)	
Unmarried	6 (3.85%)	6 (3.59%)	12 (3.72%)	
Family History of Diabetes				0.8223
Yes	69 (44.2%)	71 (42.5%)	140 (43.3%)	
No	87 (55.8%)	96 (57.5%)	183 (56.7%)	
Substance Use				0.0459
Yes	43 (27.6%)	30 (18.0%)	73 (22.6%)	
No	113 (72.4%)	137 (82.0%)	250 (77.4%)	
Education				0.0077
Literate	111 (71.2%)	94 (56.3%)	205 (63.5%)	
Illiterate	45 (28.8%)	73 (43.7%)	118 (36.5%)	
Occupation				0.0308
Employed	98 (62.8%)	124 (74.3%)	222 (68.7%)	
Unemployed	58 (37.2%)	43 (25.7%)	101 (31.3%)	
Socioeconomic Status				0.0324
Middle	43 (27.6%)	29 (17.4%)	72 (22.3%)	
Lower	113 (72.4%)	138 (82.6%)	251 (77.7%)	
CKD Stage				0.3724
Stage III	48 (30.8%)	63 (37.7%)	111 (34.4%)	
Stage II	65 (41.7%)	66 (39.5%)	131 (40.6%)	
Stage I	43 (27.6%)	38 (22.8%)	81 (25.1%)	
HTN Stage				0.6244
Stage I HTN	40 (25.6%)	36 (21.6%)	76 (23.5%)	
Stage II HTN	23 (14.7%)	33 (19.8%)	56 (17.3%)	
Prehypertension	43 (27.6%)	46 (27.5%)	89 (27.6%)	
Normal	50 (32.1%)	52 (31.1%)	102 (31.6%)	
BMI Categories				0.6529
Overweight	61 (39.1%)	59 (35.3%)	120 (37.2%)	
Obese	32 (20.5%)	41 (24.6%)	73 (22.6%)	
Normal	63 (40.4%)	67 (40.1%)	130 (40.2%)	
Co-morbidities				0.6431
Yes	53 (34.0%)	61 (36.5%)	114 (35.3%)	
No	103 (66.0%)	106 (63.5%)	209 (64.7%)	
Duration of diabetes				0.3743
Less than 10 years	79 (50.6%)	89 (53.3%)	168 (52.0%)	
More than 10 years	77 (49.4%)	78 (46.7%)	155 (48.0%)	
Duration of HTN				
Less than 5 years	44 (63.76%)	37 (58.73%)	81 (61.36%)	0.5942
More than 5 years	25 (36.23%)	26 (41.26%)	51 (38.63%)	

CKD: Chronic Kidney Disease; HTN: Hypertension; BMI: Body Mass Index.

**Table 2:** Knowledge scores among the patients stratified on the basis of demographic and clinical characteristics.

Variables	Less than 4 Correct Answers	At least 4 Correct Answers	Total	*p* Value
Age Groups				0.5832
≥50	153 (60.2%)	39 (56.5%)	192 (59.4%)	
<50	101 (39.8%)	30 (43.5%)	131 (40.6%)	
Gender				0.2231
Female	136 (53.5%)	31 (44.9%)	167 (51.7%)	
Male	118 (46.5%)	38 (55.1%)	156 (48.3%)	
Family History of Diabetes				0.1018
Yes	104 (40.9%)	36 (52.2%)	140 (43.3%)	
No	150 (59.1%)	33 (47.8%)	183 (56.7%)	
Substance Use				0.8724
Yes	57 (22.4%)	16 (23.2%)	73 (22.6%)	
No	197 (77.6%)	53 (76.8%)	250 (77.4%)	
Education				0.004
Literate	151 (59.44%)	54 (78.26%)	205 (63.5%)	
Illiterate	103 (40.56%)	15 (21.74%)	118 (36.5%)	
Occupation				0.0783
Employed	181 (71.3%)	41 (59.4%)	222 (68.7%)	
Unemployed	73 (28.7%)	28 (40.6%)	101 (31.3%)	
Socioeconomic Status				0.0003
Middle	45 (17.7%)	27 (39.1%)	72 (22.3%)	
Lower	209 (82.3%)	42 (60.9%)	251 (77.7%)	
Monthly Family Income				0.0034
More than 20,000 INR	34 (13.4%)	20 (29.0%)	54 (16.7%)	
Less than 20,000 INR	220 (86.6%)	49 (71.0%)	269 (83.3%)	
CKD Stages				0.5453
Stage III	89 (35.0%)	22 (31.9%)	111 (34.4%)	
Stage II	99 (39.0%)	32 (46.4%)	131 (40.6%)	
Stage I	66 (26.0%)	15 (21.7%)	81 (25.1%)	
HTN Stages				0.4704
Stage I HTN	63 (24.8%)	13 (18.8%)	76 (23.5%)	
Stage II HTN	40 (15.7%)	16 (23.2%)	56 (17.3%)	
Prehypertension	70 (27.6%)	19 (27.5%)	89 (27.6%)	
Normal	81 (31.9%)	21 (30.4%)	102 (31.6%)	
Glycemic Control				0.1321
Poor (HbA1c ≥ 7)	187 (73.6%)	44 (63.8%)	231 (71.5%)	
Good (HbA1c < 7)	67 (26.4%)	25 (36.2%)	92 (28.5%)	
BMI Categories				0.5314
Overweight	97 (38.2%)	23 (33.3%)	120 (37.2%)	
Obese	59 (23.2%)	14 (20.3%)	73 (22.6%)	
Normal	98 (38.6%)	32 (46.4%)	130 (40.2%)	
Co-morbidities				0.4791
Yes	87 (34.3%)	27 (39.1%)	114 (35.3%)	
No	167 (65.7%)	42 (60.9%)	209 (64.7%)	
Duration of Diabetes				0.3418
Less than 10 years	136 (53.5%)	32 (46.4%)	168 (52.0%)	
More than 10 years	118 (46.5%)	37 (53.6%)	155 (48.0%)	
Duration of HTN				
Less than 5 years	66 (62.85%)	15 (55.55%)	81 (61.36%)	0.5123
More than 5 years	39 (37.15%)	12 (44.45%)	51 (38.63%)	

INR: Indian National Rupees; CKD: Chronic Kidney Disease; HbA1c: Glycated Hemoglobin; HTN: Hypertension; BMI: Body Mass Index.

**Table 3:** Association between different domains of knowledge and demographic characteristics.

Variables	C_Q1 (Anatomy)	IC_Q1 (Anatomy)	Odds Ratio	C_Q2 (Physiology)	IC_Q2 (Physiology)	Odds Ratio	C_Q3 (Etiology)	IC_Q3 (Etiology)	Odds Ratio
Age Groups									
≥50	66 (20.4%)	126 (39.0%)	0.796 (0.503, 1.260)	80 (24.8%)	112 (34.7%)	0.771 (0.493, 1.205)	154 (47.7%)	38 (11.8%)	1.536 (0.911, 2.590)
<50	52 (16.1%)	79 (24.5%)	Reference	63 (19.5%)	68 (21.1%)	Reference	95 (29.4%)	36 (11.1%)	Reference
Gender									
Female	60 (18.5%)	107 (33.1%)	0.947 (0.602, 1.491)	70 (21.7%)	97 (30.0%)	0.821 (0.529, 1.274)	126 (39.0%)	41 (12.7%)	0.825 (0.490, 1.389)
Male	58 (18.0%)	98 (30.3%)	Reference	73 (22.6%)	83 (25.7%)	Reference	123 (38.1%)	33 (10.2%)	Reference
Family History of Diabetes									
Yes	56 (17.3%)	84 (26.0%)	1.301 (0.825, 2.053)	72 (22.3%)	68 (21.1%)	1.670 (1.070, 2.607)	115 (35.6%)	25 (7.74%)	1.682 (0.978, 2.893)
No	62 (19.2%)	121 (37.5%)	Reference	71 (22.0%)	112 (34.7%)	Reference	134 (41.5%)	49 (15.2%)	Reference
Substance Use									
Yes	30 (9.29%)	43 (13.3%)	1.284 (0.753, 2.190)	34 (10.5%)	39 (12.1%)	1.128 (0.668, 1.903)	61 (18.9%)	12 (3.72%)	1.676 (0.847, 3.317)
No	88 (27.2%)	162 (50.2%)	Reference	109 (33.7%)	141 (43.7%)	Reference	188 (58.2%)	62 (19.2%)	Reference
Education									
Literate	74 (22.9%)	131 (40.6%)	0.950 (0.594, 1.519)	87 (26.9%)	118 (36.5%)	0.816 (0.518, 1.287)	162 (50.2%)	43 (13.3%)	1.342 (0.790, 2.281)
Illiterate	44 (13.6%)	74 (22.9%)	Reference	56 (17.3%)	62 (19.2%)	Reference	87 (26.9%)	31 (9.60%)	Reference
Occupation									
Employed	76 (23.5%)	146 (45.2%)	0.731 (0.451, 1.185)	92 (28.5%)	130 (40.2%)	0.694 (0.432, 1.113)	170 (52.6%)	52 (16.1%)	0.910 (0.517, 1.603)
Unemployed	42 (13.0%)	59 (18.3%)	Reference	51 (15.8%)	50 (15.5%)	Reference	79 (24.5%)	22 (6.81%)	Reference
Socioeconomic Status									
Middle	34 (10.5%)	38 (11.8%)	**1.779** (1.045, 3.028) *	45 (13.9%)	27 (8.36%)	**2.602** (1.516, 4.467) **	63 (19.5%)	9 (2.79%)	**2.446** (1.152, 5.196) *
Lower	84 (26.0%)	167 (51.7%)	Reference	98 (30.3%)	153 (47.4%)	Reference	186 (57.6%)	65 (20.1%)	Reference
Family Income									
More than 20,000 INR (monthly)	27 (8.36%)	27 (8.36%)	**1.956** (1.084, 3.529) *	32 (9.91%)	22 (6.81%)	**2.070** (1.142, 3.752) *	47 (14.6%)	7 (2.17%)	**2.227** (0.961, 5.162) *
Less than 20,000 INR (monthly)	91 (28.2%)	178 (55.1%)	Reference	111 (34.4%)	158 (48.9%)	Reference	202 (62.5%)	67 (20.7%)	Reference
CKD Stage									
Stage III	36 (11.1%)	75 (23.2%)	0.861 (0.471, 1.574)	46 (14.2%)	65 (20.1%)	1.269 (0.703, 2.291)	86 (26.6%)	25 (7.74%)	1.536 (0.803, 2.937)
Stage II	53 (16.4%)	78 (24.1%)	1.218 (0.687, 2.160)	68 (21.1%)	63 (19.5%)	**1.935** (1.096, 3.419) *	107 (33.1%)	24 (7.43%)	1.990 (1.042, 3.800)
Stage I	29 (8.98%)	52 (16.1%)	Reference	29 (8.98%)	52 (16.1%)	Reference	56 (17.3%)	25 (7.74%)	Reference
HTN Stage									
Stage I HTN	24 (7.43%)	52 (16.1%)	0.659 (0.353, 1.231)	31 (9.60%)	45 (13.9%)	0.908 (0.497, 1.659)	46 (14.2%)	30 (9.29%)	0.307 (0.153, 0.614) **
Stage II HTN	23 (7.12%)	33 (10.2%)	0.996 (0.513, 1.931)	34 (10.5%)	22 (6.81%)	**2.037** (1.049, 3.958) **	44 (13.6%)	12 (3.72%)	0.733 (0.322, 1.671)
Prehypertension	29 (8.98%)	60 (18.6%)	0.690 (0.381, 1.250)	34 (10.5%)	55 (17.0%)	0.815 (0.456, 1.455)	74 (22.9%)	15 (4.64%)	0.987 (0.461, 2.112)
Normal	42 (13.0%)	60 (18.6%)	Reference	44 (13.6%)	58 (18.0%)	Reference	85 (26.3%)	17 (5.26%)	Reference
BMI Categories									
Overweight	39 (12.1%)	81 (25.1%)	0.770 (0.458, 1.296)	54 (16.7%)	66 (20.4%)	1.152 (0.698, 1.900)	98 (30.3%)	22 (6.81%)	1.840 (1.013, 3.343)
Obese	29 (8.98%)	44 (13.6%)	1.055 (0.586, 1.897)	35 (10.8%)	38 (11.8%)	1.296 (0.728, 2.308)	59 (18.3%)	14 (4.33%)	1.741 (0.869, 3.486)
Normal	50 (15.5%)	80 (24.8%)	Reference	54 (16.7%)	76 (23.5%)	Reference	92 (28.5%)	38 (11.8%)	Reference
Co-morbidities									
Yes	43 (13.3%)	71 (22.0%)	1.082 (0.675, 1.736)	51 (15.8%)	63 (19.5%)	1.030 (0.650, 1.630)	87 (26.9%)	27 (8.36%)	0.935 (0.545, 1.605)
No	75 (23.2%)	134 (41.5%)	Reference	92 (28.5%)	117 (36.2%)	Reference	162 (50.2%)	47 (14.6%)	Reference
Duration of Diabetes									
More than 10 years	56 (17.3%)	99 (30.7%)	0.967 (0.615, 1.522)	68 (21.1%)	87 (26.9%)	0.969 (0.625, 1.504)	117 (36.2%)	38 (11.8%)	0.840 (0.500, 1.412)
Less than 10 years	62 (19.2%)	106 (32.8%)	Reference	75 (23.2%)	93 (28.8%)	Reference	132 (40.9%)	36 (11.1%)	Reference
Glycemic Control									
Poor (HbA1c ≥ 7)	76 (23.5%)	155 (48.0%)	**1.713** (1.046, 2.806) *	95 (29.4%)	136 (42.1%)	1.562 (0.961, 2.539)	174 (53.9%)	57 (17.6%)	1.445 (0.789, 2.648)
Good (HbA1c < 7)	42 (13.0%)	50 (15.5%)	Reference	48 (14.9%)	44 (13.6%)	Reference	75 (23.2%)	17 (5.26%)	Reference
Duration of HTN #									
More than 5 years	22 (16.66%)	29 (21.97%)	1.16 (0.57, 2.36)	28 (21.21%)	23 (17.42%)	1.13 (0.55, 2.28)	39 (29.54%)	12 (9.09%)	1.28 (0.57, 2.88)
Less than 5 years	32 (24.24%)	49 (37.12%)	Reference	42 (31.81%)	39 (29.54%)	Reference	58 (43.94%)	23 (17.42%)	Reference

C: Correct response; IC: Incorrect response; INR: Indian National Rupees; CKD: Chronic Kidney Disease; HTN: Hypertension; BMI: Body Mass Index. Bold data represents significant value. * Represents significant findings (*p* < 0.05); ** Represents significant findings (*p* <0.001); # Analysis based on patients belongs to stage I and stage II HTN class only.

**Table 4:** Association between different domains of knowledge and demographic characteristics (continued).

Variables	C_Q4 (Symptoms)	IC_Q4 (Symptoms)	Odds Ratio	C_Q5 (Progression)	IC_Q5 (Progression)	Odds Ratio	C_Q6 (Treatment)	IC_Q6 (Treatment)	Odds Ratio	C_Q7 (Resource)	IC_Q7 (Resource)	Odds Ratio
Age Groups												
≥50	54 (16.7%)	138 (42.7%)	0.829 (0.511, 1.344)	23 (7.12%)	169 (52.3%)	0.802 (0.418, 1.541)	23 (7.12%)	169 (52.3%)	0.674 (0.358, 1.269)	26 (8.05%)	166 (51.4%)	0.664 (0.364, 1.211)
<50	42 (13.0%)	89 (27.6%)	Reference	19 (5.88%)	112 (34.7%)	Reference	22 (6.81%)	109 (33.7%)	Reference	25 (7.74%)	106 (32.8%)	Reference
Gender												
Female	46 (14.2%)	121 (37.5%)	0.806 (0.500, 1.300)	26 (8.05%)	141 (43.7%)	1.613 (0.829, 3.137)	20 (6.19%)	147 (45.5%)	0.713 (0.378, 1.343)	25 (7.74%)	142 (44.0%)	0.880 (0.484, 1.601)
Male	50 (15.5%)	106 (32.8%)	Reference	16 (4.95%)	140 (43.3%)	Reference	25 (7.74%)	131 (40.6%)	Reference	26 (8.05%)	130 (40.2%)	Reference
Family History of Diabetes												
Yes	48 (14.9%)	92 (28.5%)	1.467 (0.908, 2.371)	25 (7.74%)	115 (35.6%)	**2.123** (1.097, 4.109) *	21 (6.50%)	119 (36.8%)	1.169 (0.621, 2.200)	22 (6.81%)	118 (36.5%)	0.990 (0.541, 1.811)
No	48 (14.9%)	135 (41.8%)	Reference	17 (5.26%)	166 (51.4%)	Reference	24 (7.43%)	159 (49.2%)	Reference	29 (8.98%)	154 (47.7%)	Reference
Substance Use												
Yes	22 (6.81%)	51 (15.8%)	1.026 (0.581, 1.812)	13 (4.02%)	60 (18.6%)	1.651 (0.809, 3.371)	6 (1.86%)	67 (20.7%)	0.485 (0.197, 1.195)	10 (3.10%)	63 (19.5%)	0.809 (0.384, 1.707)
No	74 (22.9%)	176 (54.5%)	Reference	29 (8.98%)	221 (68.4%)	Reference	39 (12.1%)	211 (65.3%)	Reference	41 (12.7%)	209 (64.7%)	Reference
Education												
Literate	64 (19.8%)	141 (43.7%)	1.220 (0.738, 2.015)	27 (8.36%)	178 (55.1%)	1.041 (0.530, 2.048)	28 (8.67%)	177 (54.8%)	0.940 (0.490, 1.801)	28 (8.67%)	177 (54.8%)	0.653 (0.357, 1.197)
Illiterate	32 (9.91%)	86 (26.6%)	Reference	15 (4.64%)	103 (31.9%)	Reference	17 (5.26%)	101 (31.3%)	Reference	23 (7.12%)	95 (29.4%)	Reference
Occupation												
Employed	61 (18.9%)	161 (49.8%)	0.714 (0.431, 1.184)	25 (7.74%)	197 (61.0%)	0.627 (0.322, 1.222)	33 (10.2%)	189 (58.5%)	1.295 (0.638, 2.626)	30 (9.29%)	192 (59.4%)	0.595 (0.322, 1.102)
Unemployed	35 (10.8%)	66 (20.4%)	Reference	17 (5.26%)	84 (26.0%)	Reference	12 (3.72%)	89 (27.6%)	Reference	21 (6.50%)	80 (24.8%)	Reference
Socioeconomic Status												
Middle	31 (9.60%)	41 (12.7%)	**2.164** (1.254, 3.733) **	15 (4.64%)	57 (17.6%)	**2.183** (1.090, 4.374) *	11 (3.41%)	61 (18.9%)	1.151 (0.551, 2.405)	21 (6.50%)	51 (15.8%)	**3.033** (1.607, 5.726) **
Lower	65 (20.1%)	186 (57.6%)	Reference	27 (8.36%)	224 (69.3%)	Reference	34 (10.5%)	217 (67.2%)	Reference	30 (9.29%)	221 (68.4%)	Reference
Family Income												
More than 20,000 INR (monthly)	23 (7.12%)	31 (9.60%)	**1.992** (1.091, 3.640) *	13 (4.02%)	41 (12.7%)	**2.624** (1.260, 5.463) **	10 (3.10%)	44 (13.6%)	1.519( 0.701, 3.292)	15 (4.64%)	39 (12.1%)	**2.489** (1.247, 4.969) **
Less than 20,000 INR (monthly)	73 (22.6%)	196 (60.7%)	Reference	29 (8.98%)	240 (74.3%)	Reference	35 (10.8%)	234 (72.4%)	Reference	36 (11.1%)	233 (72.1%)	Reference
CKD Stage												
Stage III	27 (8.36%)	84 (26.0%)	0.811 (0.424, 1.551)	8 (2.48%)	103 (31.9%)	**0.292** (0.119, 0.717) *	16 (4.95%)	95 (29.4%)	1.537 (0.624, 3.787)	16 (4.95%)	95 (29.4%)	0.968 (0.431, 2.177)
Stage II	46 (14.2%)	85 (26.3%)	1.365 (0.748, 2.491)	17 (5.26%)	114 (35.3%)	0.561 (0.268, 1.175)	21 (6.50%)	110 (34.1%)	1.742 (0.732, 4.143)	23 (7.12%)	108 (33.4%)	1.225 (0.572, 2.620)
Stage I	23 (7.12%)	58 (18.0%)	Reference	17 (5.26%)	64 (19.8%)	Reference	8 (2.48%)	73 (22.6%)	Reference	12 (3.72%)	69 (21.4%)	Reference
HTN Stage												
Stage I HTN	19 (5.88%)	57 (17.6%)	0.926 (0.469, 1.829)	11 (3.41%)	65 (20.1%)	1.400 (0.572, 3.424)	11 (3.41%)	65 (20.1%)	1.159 (0.488, 2.750)	11 (3.41%)	65 (20.1%)	1.064 (0.454, 2.494)
Stage II HTN	20 (6.19%)	36 (11.1%)	1.543 (0.765, 3.113)	10 (3.10%)	46 (14.2%)	1.798 (0.712, 4.544)	10 (3.10%)	46 (14.2%)	1.489 (0.607, 3.654)	11 (3.41%)	45 (13.9%)	1.537 (0.645, 3.659)
Prehypertension	30 (9.29%)	59 (18.3%)	1.412 (0.758, 2.630)	10 (3.10%)	79 (24.5%)	1.047 (0.422, 2.596)	11 (3.41%)	78 (24.1%)	0.965 (0.409, 2.278)	15 (4.64%)	74 (22.9%)	1.274 (0.578, 2.811)
Normal	27 (8.36%)	75 (23.2%)	Reference	11 (3.41%)	91 (28.2%)	Reference	13 (4.02%)	89 (27.6%)	Reference	14 (4.33%)	88 (27.2%)	Reference
BMI Categories												
Overweight	29 (8.98%)	91 (28.2%)	0.623 (0.358, 1.084)	16 (4.95%)	104 (32.2%)	0.755( 0.376, 1.518)	18 (5.57%)	102 (31.6%)	1.031 (0.513, 2.073)	12 (3.72%)	108 (33.4%)	0.424 (0.204, 0.881)
Obese	23 (7.12%)	50 (15.5%)	0.899 (0.487, 1.660)	4 (1.24%)	69 (21.4%)	**0.285** (0.094, 0.861) *	8 (2.48%)	65 (20.1%)	0.719 (0.298, 1.735)	12 (3.72%)	61 (18.9%)	0.750 (0.354, 1.589)
Normal	44 (13.6%)	86 (26.6%)	Reference	22 (6.81%)	108 (33.4%)	Reference	19 (5.88%)	111 (34.4%)	Reference	27 (8.36%)	103 (31.9%)	Reference
Co-morbidities												
Yes	35 (10.8%)	79 (24.5%)	1.075 (0.654, 1.767)	18 (5.57%)	96 (29.7%)	1.445 (0.748, 2.794)	15 (4.64%)	99 (30.7%)	0.904 (0.464, 1.761)	21 (6.50%)	93 (28.8%)	1.347 (0.731, 2.483)
No	61 (18.9%)	148 (45.8%)	Reference	24 (7.43%)	185 (57.3%)	Reference	30 (9.29%)	179 (55.4%)	Reference	30 (9.29%)	179 (55.4%)	Reference
Duration of Diabetes												
More than 10 years	51 (15.8%)	104 (32.2%)	1.340 (0.831, 2.163)	21 (6.50%)	134 (41.5%)	1.097 (0.574, 2.098)	22 (6.81%)	133 (41.2%)	1.043 (0.555, 1.958)	26 (8.05%)	129 (39.9%)	1.153 (0.634, 2.097)
Less than 10 years	45 (13.9%)	123 (38.1%)	Reference	21 (6.50%)	147 (45.5%)	Reference	23 (7.12%)	145 (44.9%)	Reference	25 (7.74%)	143 (44.3%)	Reference
Glycemic Control												
Poor (HbA1c ≥ 7)	69 (21.4%)	162 (50.2%)	0.975 (0.574, 1.657)	32 (9.91%)	199 (61.6%)	0.758 (0.356, 1.614)	34 (10.5%)	197 (61.0%)	0.787 (0.380, 1.629)	39 (12.1%)	192 (59.4%)	0.739 (0.368, 1.484)
Good (HbA1c < 7)	27 (8.36%)	65 (20.1%)	Reference	10 (3.10%)	82 (25.4%)	Reference	11 (3.41%)	81 (25.1%)	Reference	12 (3.72%)	80 (24.8%)	Reference
Duration of HTN #												
More than 5 years	14 (10.60%)	37 (27.81%)	0.75 (0.35, 1.63)	12 (9.09%)	40 (30.30%)	0.857 (0.379, 1.935)	8 (6.06%)	43 (32.57%)	0.890 (0.344, 2.300)	4 (3.03%)	47 (65.60%)	0.899 (0.249, 3.241)
Less than 5 years	27 (20.45%)	54 (40.90%)	Reference	21 (15.90%)	60 (45.45%)	Reference	14 (10.60%)	67 (50.75%)	Reference	7 (5.30%)	74 (56.06%)	Reference

C: Correct response; IC: Incorrect response; INR: Indian National Rupees; CKD: Chronic Kidney Disease; HTN: Hypertension; BMI: Body Mass Index. Bold data represents significant value. * Represents significant findings (*p* < 0.05); ** Represents significant findings (*p* <0.001); # Analysis based on patients belongs to stage I and stage II HTN class only.

## Supplementary Materials

The following are available online at https://www.mdpi.com/1660-4601/16/8/1443/s1: Supplementary File S1: Questionnaire to assess the awareness of kidney disease.
