# Supplementary material for: Limited Knowledge of Chronic Kidney Disease among Type 2 Diabetes Mellitus Patients in India
Source: Int J Environ Res Public Health. 2019 Apr 23;16(8):1443. doi: 10.3390/ijerph16081443 (PMC6518175; doi:10.3390/ijerph16081443)
Supplement: Supplementary file 1 [file ijerph-16-01443-s001.pdf]

Supplementary File S1

Questionnaire to assess the awareness of kidney disease:

1. How many healthy kidney(s) does a person need to lead a normal life?
   ① One ② Two ③ I don't know
2. What is the function of a kidney in a human body?
   ① To break down food
   ② To produce substances that breakdown fat
   ③ To filter waste products in the blood
   ④ I don't know
3. What can cause kidney disease?
   ① High blood pressure
   ② Diabetes
   ③ Inherited condition
   ④ All of the above
   ⑤ I don't know
4. What are the symptoms of early kidney disease that might progress to kidney failure?
   ① Bubbles in the urine
   ② Back pain
   ③ Blood in the urine
   ④ Can present without any symptoms/ complaints
   ⑤ All of the above
   ⑥ I don't know
5. Which of the following statement about kidney disease is INCORRECT:
   ① Kidney disease can be prevented
   ② Kidney disease can be cured with medications
   ③ A person is said to have kidney disease when he/she needs dialysis
   ④ None of the above
   ⑤ I don't know
6. Where can dialysis treatment be carried out?
   ① In a dialysis centre and at home
   ② Only in a dialysis centre
   ③ Only at home
   ④ I don't know
7. What is the best medical treatment for End Stage Kidney Failure?
   ① Medication
   ② Dialysis
   ③ Kidney transplant
   ④ I don't know
